# Effects of a comprehensive intervention on hypertension control in Chinese employees working in universities based on mixed models

**DOI:** 10.1038/s41598-019-55849-6

**Published:** 2019-12-16

**Authors:** Yang Li, Jin Xiaoqing, Tang Xinhua, Shou Xiaoling, Xu Xiaoling, Yu Wei, Wang Zengwu, Wang Xin, Zheng Pinpin, Yan Jing

**Affiliations:** 1 Zhejiang Provincial Center for Cardiovascular Disease Control and Prevention, Zhejiang Hospital, Hangzhou, 310013 China; 2 Key Laboratory of Public Health Safety, Ministry of Education, Health Communication Institute, Fudan University, 138 Yixueyuan Road, Shanghai, 200032 China; 3 Chinese Acupuncture Department, Zhejiang Hospital, Hangzhou, 310013 China; 4 Department of Community Prevention and Control, National Cardiovascular Disease Center, Fuwai Hospital, BeiJing, 102308 China

**Keywords:** Public health, Clinical trial design

## Abstract

We conducted a comprehensive intensive intervention for hypertension patients working in universities or colleges. From July 2015 to March in 2016, 220 hypertension subjects were recruited, with 165 cases in intensive intervention group and 55 in standard intervention group. After 24 months of intervention, 208 ones including of 157 in intensive intervention group and 51 in standard intervention group were included in the final analysis. The patients in standard intervention group were given routine intervention, which mainly including of drug treatment and health education. The patients in intervention group were given comprehensive intensive intervention in addition to routine intervention, including follow-up management of hypertension, emotional, lifestyle intervention and else. The study and experimental protocols were approved by institutional review board of Zhejiang Hospital and Fu Wai Hospital and registered (ChiCTR-ECS-14004641, date of registration: May 8, 2014). After 2 years, compared with the standard intervention group, SBP/DBP in the intensive intervention group decreased by 3.7/4 mmHg and BP control rate increased by 8.9%, and the unhealthy behaviors and life quality including tension and pressure were also improved in the intensive intervention group. We used mixed effect model to analyze the intervention effect which could solve the problems of missing values and correlation. The intensive intervention of hypertension control including follow-up management, emotional and lifestyle intervention in occupational places could promote the development of the prevention, treatment and control of hypertension among staff in colleges and universities.

## Introduction

Cardiovascular disease has become an important cause of death, and the related medical expense has brought great economic burden to the country and individuals. Hypertension is an important risk factor for cardiovascular and cerebrovascular diseases such as stroke and coronary heart disease^1,2^. Many studies have found that anxiety, depression are correlated with high blood pressure (BP)^3,4^, and patients with hypertension are more anxious which leading a high BP.

The prevalence of hypertension among Chinese adults aged 18 and over is 23.2%, and treatment rate and control rate is 40.7% and 15.3%, respectively. And the rates are 26.5%, 18.9% and 6.5% respectively among occupational population in China, indicating that the prevalence of hypertension among employees is similar to the national level, but the treatment and control status are poor^5–7^. Most adults aged 30–60 are belonged to occupational groups, they spend about 1/3 of their waking hours in work places. Their health is closely related to social stability and development. With the development of the social economy, occupational groups are more prone to anxiety and depression^8^, and working population with ideal BP are less than 1/3 in China. Among the occupational population, college employees, mainly intellectuals, have been responsible for teaching and scientific research for long time. Many comprehensive factors, such as heavy tasks, mental tension, psychological pressure, lack of exercise and neglect of health care, lead to high BP, making them vulnerable to hypertension. But there were few studies focusing on this group of working population in China. And the work of prevention and treatment of cardiovascular disease among occupational population is relatively weak, while hypertension management in urban communities does not include occupational population in the workplaces.

In the present study, we aimed to provide a suitable way to control hypertension for occupational population, and evaluate the comprehensive intensive intervention for hypertension patients working in universities or colleges.

## Methods

### Study design and population

Using the method of multistage stratified random sampling, schools that were not medical ones with more than 500 staff in Zhejiang province and wanted to participant were recruited in January, 2013. And 4 schools with similar scale were selected from 20 ones randomly, they were randomly assigned to intensive intervention group (3 school) and standard intervention group (1 school) respectively, making more participants to receive more comprehensive intervention. Hypertension subjects were screened and diagnosed among the 4 schools, and 55 subjects were selected from every school randomly. Completely random grouping method was used. The statistician generated the random allocation sequence and assigned schools to different groups, and the participants were enrolled by physicians and nurses randomly. Random Numbers were generated by the statistician using computer software (SAS 9.4).

In order to reduce bias, double blinding design was adopted in this study, physicians, nurses and participants didn’t know which group the subjects were assigned to. When the subject had a serious adverse event or was in urgent need of rescue, then we could break blindness and opened the emergency letter, the cause and time should be recorded and signed for confirmation. And if the total breaking blinding rate or the rate of opening emergency letters exceeded 20%, which meaning that the double-blinding study failed, then another new study needed to be arranged.

In this study, the primary outcomes included hypertension control rate and BP, and secondary outcomes included lifestyle changes, quality of life and relative biochemical variables changes. Random effect optimization model and the sample size calculation formula for the rate comparison between two groups were used to calculate the sample size, the expected effect size of the difference of the hypertension control rate changes between the intensive intervention group and the standard intervention group was set as 10%, test level was 0.05, power was 98%. The sample size was calculated to be about 50 cases, and the final sample size was 165 cases in the intensive intervention group and 55 cases in the standard intervention group respectively, which taking into account the problem of follow-up lost, etc.

Subjects with hypertension, employed in selected schools and signed informed consents were included in this study. Hypertension were defined as systolic blood pressure (SBP) equaled to 140 mm Hg or more and/or diastolic blood pressure (DBP) equaled to 90 mmHg or more with BP measured 3 times on different days, or having a history of hypertension or taking antihypertensive drugs within 2 weeks^9^. Patients were excluded if she/he had secondary hypertension, acute myocardial infarction (AMI, diagnosis in 3 months), acute stroke (diagnosis in 6 months), severe illnesses, and who were pregnant and lactating women or medical staff. Criteria for suspension: Subjects who were unwilling to continue to participate in the study, patients with severe physical diseases after enrollment, and other reasons for violation of this study, such as leaving schools, etc.

**Figure 1:**
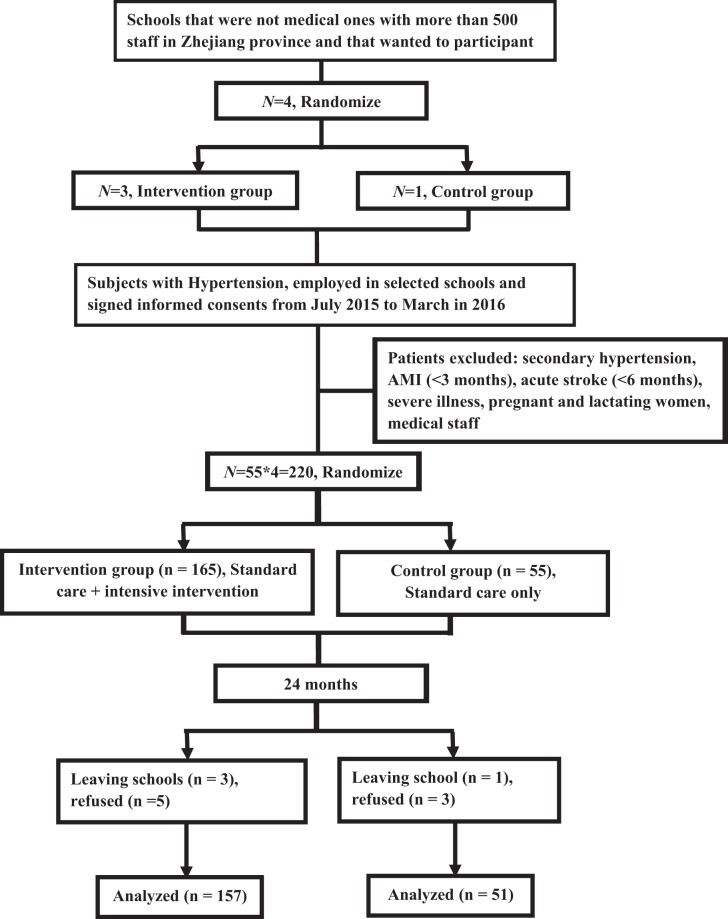
Flowchart of enrollment of participants in the study.

From July 2015 to March in 2016, 220 subjects were recruited, with 165 cases in intensive intervention group and 55 in standard intervention group, respectively. After 24 months of intervention, 8 cases were excluded from intensive intervention group: 3 left schools, and 5 refused to continue. 4 cases were excluded from standard intervention group: 1 left school, 3 refused to continue. A total of 208 subjects were included in the final analysis, 157 in intensive intervention group and 51 in standard intervention group (response rate was 94.5%) (Fig. 1).

### Ethical considerations

The protocol of this study was approved by the Medical Ethics Committee of Zhejiang hospital and Institutional Review Board of the Fu Wai Cardiovascular Hospital (approval no. 2012–402, approval date, 17 July 2012). The participants were informed about the objectives and methods of the study. They were informed that their participation was totally voluntary and that they could withdraw from the study at any time without citing any reason. Written and signed or thumb printed informed consent was obtained from those who agreed to participate. The study and experimental protocols were approved by institutional review board of Zhejiang hospital and Fu Wai Hospital and registered (ChiCTR-ECS-14004641, date of registration: May 8, 2014). All methods were performed in accordance with the relevant guidelines and regulations. The conduct of the study was monitored by an independent data and safety monitoring committee.

### Intervention

According to the Chinese guidelines for prevention and control of hypertension in 2010, and the policy statement on implementing workplace health programs to prevent cardiovascular disease issued by the American heart association (AHA)^9,10^, we developed the comprehensive intensive intervention for hypertension control, which also considering of the actual situation in the workplace.

The comprehensive intensive intervention included: (1) Risk stratification, stratified management and follow-up management of hypertension^9^. (2) Standard medication treatment and non-medicine treatment^9^. (3) Education intervention aiming to enhance understanding and knowledge about hypertension of patients. (4) Emotional intervention aiming to help patients deal with negative emotions, such as anxiety, depression and others. (5) Lifestyle interventions including education of healthy habits, creating healthy environment such as opening the windows for low salt and low fat food, smoking control in the workplace, providing services such as body weight measurement, body fat measurement and BP measurement, implementing work exercise project, and carrying out regular physical examination.

The standard intervention conducted in control group included basic drug treatment and simple health education.

We conducted a 2-year comprehensive intensive intervention for employees with hypertension working in schools of China. We measured the effects of intervention on their BP and BP control rate, lifestyles, quality of life, etc., and compared the results with those of a standard intervention group that conducting conventional care.

### Data collection and assessment

All hypertensive participants were followed-up at 6th month, 1th year, and 2th year after the initiation of the study. BP, questionnaire information such as baseline characteristics, lifestyles, life quality information, physical examination and laboratory examination were collected. The intensive intervention group collected some information monthly to get dynamic changes of some major variables including BP, BP control rate and lifestyles, such as smoking, drinking, fruit consumption, vegetable consumption, exercise and else.

BP was measured using a standard mercury sphygmomanometer. The subjects were asked to rest for 5 mins in a sitting position, after which the BP in their right upper arm was measured twice, with the measurements at least 1 min apart. SBP and DBP were recorded as phase I and V Korotkoff sounds. If the difference between the two measurements exceeded 5 mm Hg (1 mm Hg = 0.133 kPa), the BP was measured again. The average BP was then recorded^1^.

Life quality of patients was measured with the Spanish Hypertension Quality of Life Questionnaire (MINICHAL). We chose the MINICHAL because it could be completed in a short time and evaluate the quality of life relatively comprehensively. MINICHAL evaluation including mental status (ten questions) and somatic manifestations (six questions)^1^. Results closer to zero indicated better quality of life^11^. The questionnaire is shown in supplement 1.

Height, weight and other physical examination and laboratory examination were measured by trained medical staff in accordance with the standard protocol^12^. Smoking was defined as smoking 1 cigarette per day at least, or cumulative smoking for 6 months, drinking was defined as drinking once or more per week, regular exercise was defined as exercising 3 times or more per week and lasting 30 mins or more per time.

### Statistical analysis

Epidata 3.0 was used for data entry and validation and SAS 9.4 for data management and analysis. Sociodemographic characteristics of participants were summarized using frequencies (percentages) or means and standard deviations, and they were compared by the Student’s t-test and χ^2^ test, respectively.

We used mixed models in our analyses to see how outcomes changed over time and how they were affected by intervention. The model included fixed effects for intervention groups, time of measurement, and the model were adjusted by some baseline characteristics such as age, gender, educational level, and so on. An interaction term between intensive intervention group/standard intervention group and time was also included to determine if the 2 interventions led to different recovery trajectories over time. In addition, the model included a random effect for the baseline values, addressing the variability in the starting point for each patient. Significance level was set at *P* < 0.05 for all hypothesis tests.

We used the results of per-protocol analysis method in this study, considering the slight difference between the results of the intention-to-treat and per-protocol analysis approaches.

## Results

### Baseline characteristics of the study participants

208 subjects were included in this study, including 157 in the intensive intervention group and 51 in the standard intervention group. The mean age of the total population was 50.1 years old, 50.6 years old in the intensive intervention group and 48.5 years old in the standard intervention group. There were 53 females, accounting for 25.5% of the total population, 32 in the intensive intervention group (26.8%) and 11 in the standard intervention group (21.6%). The differences of diabetes history, family history of hypertension and stroke, smoking status, exercise status, fasting blood glucose (FBG) and serum creatinine (SC) between the 2 groups were statistically significant (P < 0.05) (Table 1).

**Table 1:** Baseline Characteristics of the Study Participants.

Variable	Overall (n = 208)	Intensive group1 (n = 53)	Intensive group2 (n = 50)	Intensive group3 (n = 54)	Intensive group (n = 157)	Standard group (n = 51)	Difference*	P Value^†^
Age, y	50.1 ± 7.2	50.49 ± 7.8	50.38 ± 5.5	50.94 ± 4.9	50.6 ± 5.4	48.5 ± 9.9	2.1	0.18
**Age categories, y**								0.23
<Median of 50	97 (46.6)	18 (34.0)	24 (48.0)	19 (35.2)	61 (38.9)	15 (36.4)	15 (2.5)	
≥Median of 50	111 (53.4)	35 (66.0)	26 (52.0)	35 (64.8)	96 (61.1)	36 (63.6)	60 (−2.5)	
Female sex	53 (25.5)	12 (22.6)	14 (28.0)	16 (29.6)	42 (26.8)	11 (21.6)	31 (0.2)	0.45
Education								0.31
University and above	160 (76.9)	36 (67.9)	43 (86.0)	45 (83.3)	124 (79.0)	39 (76.5)	85 (2.5)	
High school and below	48 (23.1)	17 (32.1)	7 (14.0)	9 (16.7)	33 (21.0)	12 (23.5)	21 (−2.5)	
**History of cardiovascular disease**								
Hypertension	203 (97.6)	52 (98.1)	50 (100)	50 (92.6)	154 (98.1)	49 (96.1)	105 (2.0)	0.17
Dyslipidemia	76 (36.5)	20 (37.7)	24 (48.0)	15 (27.8)	59 (37.6)	17 (33.3)	42 (4.3)	0.62
Diabetes	19 (9.13)	5 (9.43)	6 (12.0)	8 (14.8)	19 (12.1)	0 (0.0)	19 (12.1)	**0.003**
Myocardial infarction	1 (0.48)	0 (0.00)	0 (0.00)	0 (0.00)	0 (0.00)	1 (1.96)	−1 (−1.96)	0.09
Stroke	1 (0.48)	0 (0.0)	1 (2.0)	0 (0.0)	1 (0.64)	0 (0.0)	1 (0.64)	0.18
**Family history of cardiovascular disease**								
Hypertension	114 (54.81)	38 (71.7)	32 (64.0)	28 (51.8)	94 (59.87)	20 (39.22)	74 (20.65)	**0.004**
Stroke	13 (6.25)	3 (5.7)	5 (10.0)	5 (9.2)	13 (8.28)	0 (0.0)	13 (8.28)	**0.01**
Coronary heart disease	25 (12.0)	5 (9.4)	9 (18.0)	7 (13.0)	21 (13.4)	4 (7.8)	17 (5.6)	0.53
Diabetes	23 (11.1)	7 (13.2)	8 (16.0)	5 (9.2)	20 (12.7)	3 (5.9)	17 (6.8)	0.17
**Lifestyle habits**								
Smoking	60 (28.9)	15 (28.3)	10 (20.0)	21 (38.9)	46 (29.3)	14 (27.5)	32 (1.8)	0.8
Drinking	80 (38.5)	19 (35.8)	17 (34.0)	18 (33.3)	54(34.4)	26 (51.0)	28 (−16.6)	**0.04**
Regular exercise	113 (54.3)	24 (45.3)	25 (50.0)	30 (55.5)	79 (50.3)	34 (66.7)	45 (−16.4)	**0.04**
BMI, kg/m2	25.2 ± 2.6	25.0 ± 2.6	25.5 ± 2.7	25.5 ± 2.7	25.3 ± 2.7	24.7 ± 2.6	0.6	0.16
SBP, mm Hg	137.9 ± 13.6	134.9 ± 13.7	136.9 ± 13.9	137.7 ± 15.0	136.8 ± 13.8	141.1 ± 12.4	−4.3	**0.04**
DBP, mm Hg	87.9 ± 9.4	87.0 ± 9.5	87.9 ± 10.1	88.0 ± 9.7	87.3 ± 9.8	89.7 ± 8.0	−2.4	0.11
FBG, mmol/L	5.8 ± 1.7	5.7 ± 2.1	6.1 ± 1.8	5.9 ± 2.0	5.9 ± 1.9	5.2 ± 0.6	0.7	**<0.0001**
TC, mmol/L	5.2 ± 1.0	5.1 ± 1.0	5.3 ± 1.5	5.2 ± 1.2	5.2 ± 1.1	5.0 ± 0.9	0.2	0.29
TG, mmol/L	2.0 ± 1.6	2.2 ± 1.9	2.0 ± 2.1	1.9 ± 2.0	2.1 ± 1.8	1.7 ± 1.3	0.4	0.09
HDL-C, mmol/L	1.32 ± 0.37	1.34 ± 0.5	1.35 ± 0.6	1.31 ± 0.3	1.33 ± 0.4	1.3 ± 0.26	0.03	0.27
SC,μmol/L	85.0 ± 23.2	88.1 ± 24.9	87.3 ± 25.9	89.3 ± 26.2	88.2 ± 25.1	74.8 ± 11.2	13.4	**<0.0001**

BMI, body mass index; SBP, systolic blood pressure; DBP, diastolic blood pressure; FBG, Fasting plasma glucose; TC, Total cholesterol; TG, triglycerides; HDL-C, High density lipoprotein; SC, serum creatinine. Sociodemographic characteristics of participants were summarized by areas using frequencies (percentages) or means and standard deviations, and they were compared by Chi-squared tests and the Student’s t-test, respectively. *Test for differences between intensive group and standard group. Statistically significant results are presented in bold.

### Effects of intervention on BP and BP-Related variables

**Figure 2:**
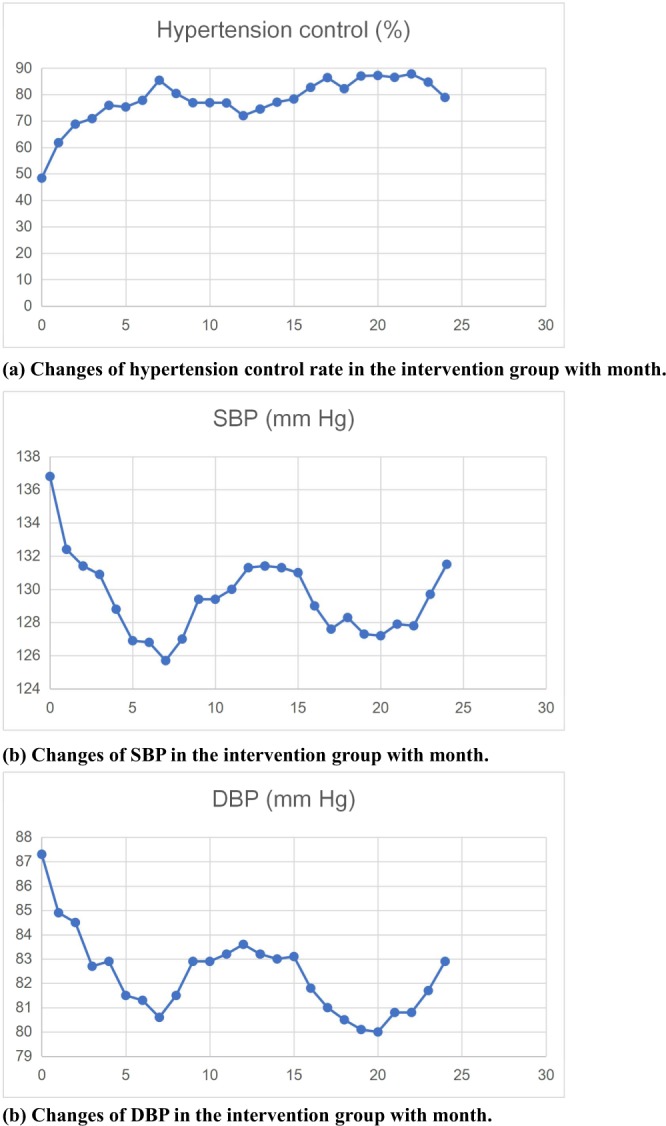
Changes of blood pressure related indexes in the intervention group with month. (**a**) Changes of hypertension control rate in the intervention group with month. (**b**) Changes of SBP in the intervention group with month. (**b**) Changes of DBP in the intervention group with month.

Compared with the baseline, the SBP/DBP of the patients in the intensive intervention group decreased by 5.3/4.4 mmHg after two years’ comprehensive intensive intervention, respectively, and the hypertension control rate increased from 48.4% to 78.9% (all, *P* < 0.05), as shown in Table 2. The difference of the hypertension control rate changes between the intervention group and the control group was 19.5% (*P* < 0.05). Meanwhile, the trend chart showed that the SBP/DBP of the patients in the intensive intervention group decreased month by month, and the hypertension control rate increased month by month (all, *P* < 0.05), as shown in Fig. 2. In addition, among the risk factors related to cardiovascular disease, vegetable consumption increased by 2 g/day, fruit consumption increased by 2.1 g/day, regular exercise increased by 1.2 times/week, smoking rate decreased by 7.5%, drinking rate decreased by 31%, and the mean score of MINICHAL decreased by 10.4 (all, *P* < 0.05). SBP in the standard intervention group decreased by 1.6 mmHg, the hypertension control rate increased from 19.6% to 41.2%, and the drinking rate decreased from 51% to 47.1% (all, *P* < 0.05). Other differences were not statistically significant.

**Table 2:** Effects of intervention on BP and BP-Related variables.

Variable, mean (SD)/N (%)	Group	Baseline	24th month	Difference*	P Value^†^
**BP**					
SBP, mm Hg	Intensive	136.8 ± 13.8	131.5 ± 9.3	**−5.3**	**<0.0001**
	Standard	141.1 ± 12.4	139.5 ± 10.5	**−1.6**	
DBP, mm Hg	Intensive	87.3 ± 9.8	82.9 ± 6.1	**−4.4**	**<0.0001**
	Standard	89.7 ± 7.9	89.3 ± 6.7	**−0.4**	
BP control	Intensive	76 (48.4)	123 (78.9)	**47 (30.5)**	**<0.0001**
	Standard	10 (19.6)	21 (41.2)	**11 (21.6)**	
**BP-related behavioral variables**					
BMI, kg/m^2^	Intensive	25.3 ± 2.6	25.0 ± 2.9	−0.3	0.675
	Standard	24.7 ± 2.5	24.8 ± 3.6	0.1	
Waistline, cm	Intensive	87.7 ± 8.5	87.1 ± 8.5	−0.6	0.365
	Standard	86.3 ± 8.2	85.9 ± 9.9	−0.4	
Vegetable use, g/day	Intensive	6.7 ± 3.0	8.7 ± 2.3	**2**	**0.0078**
	Standard	8.1 ± 4.5	7.9 ± 3.4	−0.5	
Fruit use, g/day	Intensive	2.7 ± 2.1	4.8 ± 2.9	**2.1**	**<0.0001**
	Standard	4.4 ± 3.4	4.2 ± 1.9	−0.2	
Regular exercise, times/week	Intensive	3.0 ± 2.4	4.2 ± 1.9	**1.2**	**0.0025**
	Standard	3.9 ± 2.3	4.0 ± 2.0	0.1	
Smoking	Intensive	46 (29.3)	34 (21.8)	**−12 (−7.5)**	**<0.0001**
	Standard	14 (27.4)	15 (29.4)	1 (−2)	
Drinking	Intensive	54 (34.4)	23 (14.7)	**−31 (−19.7)**	**<0.0001**
	Standard	26 (51.0)	24 (47.1)	**−2 (−3.9)**	
**Biochemical variables**					
FBG, mmol/L	Intensive	5.9 ± 1.9	5.8 ± 1.3	−0.1	0.0589
	Standard	5.2 ± 0.6	5.3 ± 0.8	0.1	
TC, mmol/L	Intensive	5.2 ± 1.0	5.1 ± 1.0	−0.1	0.6
	Standard	5.0 ± 0.9	5.1 ± 0.9	0.1	
TG, mmol/L	Intensive	2.0 ± 1.8	1.8 ± 1.1	−0.2	0.294
	Standard	1.6 ± 1.3	1.5 ± 1.3	−0.1	
HDL-C, mmol/L	Intensive	5.2 ± 1.0	5.1 ± 0.9	−0.1	0.269
	Standard	5.0 ± 0.9	5.1 ± 1.0	0.1	
SC, μmol/L	Intensive	88.2 ± 25.1	88.6 ± 16.0	−0.6	0.21
	Standard	74.8 ± 11.2	75.2 ± 10.4	0.4	
MINICHAL, score	Intensive	22.4 ± 4.8	12.0 ± 4.4	**−10.4**	**<0.0001**
	Standard	22.1 ± 4.0	21.3 ± 3.8	0.2	
Anxiety and tension	Intensive	3.5 ± 1.8	1.0 ± 0.4	**−2.5**	**<0.0001**
	Standard	3.0 ± 2.1	2.9 ± 1.8	−0.1	

BP, blood pressure; SBP, systolic blood pressure; DBP, diastolic blood pressure; FBG, Fasting plasma glucose; TC, Total cholesterol; TG, triglycerides; HDL, High density lipoprotein; SC, serum creatinine; BMI, body mass index; MINICHAL, Spanish Hypertension Quality of Life Questionnaire. Mixed models were used in the analyses to see how outcomes changed over time and how they were affected by treatment. The model included fixed effects for intervention group, time of measurement, and the model were adjusted by some baseline characteristics such as age, gender, educational level, and so on. Differences* are the differences of mean/cases between the 24th month and the baseline. Statistically significant results are presented in bold. P Values† are for differences between intensive group and standard group. Statistically significant results are presented in bold.

The results of mixed effect model analysis showed that after adjusting confounding factors, the intervention effects of SBP, DBP, hypertension control rate, vegetable consumption, fruit consumption, regular exercise, smoking rate, drinking rate and MINICHAL score were −3.7 mmHg, −4mmHg, 8.9%, 2.5 g/day, 2.3 g /day, 1.1 times/week, −5.5%, −15.8% and −10.6 points, respectively (all, *P* < 0.05) (Table 2).

### Prespecified subgroup analyses of hypertension control rate (%)

By the end of the intervention, 144 patients had their BP controlled. There were 47 participants having their BP controlled after intensive intervention that were uncontrolled at the baseline in the intensive intervention group, and 11 patients in the standard intervention group, the difference of control rate of intervention effect was 8.9% (*P* < 0.05). When the stratification variables were included, we found that the intervention had an effect on BP control in various subgroups, including the younger than 50 years old, the male, university and above, drinking and irregular exercise, and the effect was 17.7%, 32.2%, 13.1%, 16.4%, 30.2% and 13.7%, respectively (Table 3).

**Table 3:** Prespecified subgroup analyses of hypertension control effect.

Variables	Group	Baseline	24th month	Difference (95% CI)*	Intervention effect (95% CI)†
**Age, y**					
<Median of 50	Intensive	54.1	91.8	37.7 (10.7–52.8)	**17.7 (3.8–31.9)**
	Standard	53.3	73.3	20 (6.1–37.1)	
≥Median of 50	Intensive	44.8	69.8	25 (4.9–48.2)	2.7 (−4.9–18.3)
	Standard	5.5	27.8	22.3 (10.7–50.2)	
**Gender**					
Female	Intensive	71.4	95.5	24.1 (8.4–43.9)	**5.9 (0.1–24.2)**
	Standard	18.2	36.4	18.2 (8.4–30.5)	
Male	Intensive	40	72.17	32.2 (11.2–58.3)	**32.2 (8.0–43.1)**
	Standard	20	20	0	
**Education**					
University and above	Intensive	52.4	76.6	24.2 (11.5–46.3)	**13.1 (2.1–23.7)**
	Standard	16.7	27.8	11.1 (4.9–30.4)	
High school and below	Intensive	33.3	84.8	51.5 (23.6–73.1)	**4.9 (0.2–16.2)**
	Standard	26.7	73.3	46.7 (23.0–67.6)	
**Smoking status**					
No	Intensive	54.9	79.28	24.33 (9.3–36.3)	**8.1 (1.2–18.2)**
	Standard	18.9	35.14	16.22 (8.9–34.2)	
Yes	Intensive	32.6	76.09	43.48 (32.0–51.1)	**7.8 (1.0–19.8)**
	Standard	21.4	57.14	35.71 (26.2–52.2)	
**Drinking status**					
No	Intensive	52.4	85.44	33.0 (19.5–41.7)	1 (−8.1–19.2)
	Standard	12	44	32.0 (11.9–43.2)	
Yes	Intensive	40.7	64.8	24.1 (10.2–42.2)	**16.4 (3.2–31.8)**
	Standard	26.9	34.6	7.7 (−2.3–21.3)	
**Regular exercise**					
No	Intensive	52.6	76.9	24.4 (12.3–34.9)	**30.2 (11.7–45.8)**
	Standard	29.4	23.5	−5.9 (−10.2–8.8)	
Yes	Intensive	44.3	79.7	35.4 (12.9–54.9)	0.2 (−0.6–1.7)
	Standard	14.7	50	35.3 (10.5–51.2)	
**BMI, kg/m2**					
<Median of 28	Intensive	51.1	84.7	33.6 (12.7–48.3)	9.7 (−2.1–20.7)
	Standard	19.6	43.5	23.9 (9.5–45.8)	
≥Median of 28	Intensive	34.6	46.2	11.5 (−2.9–31.7)	11.5 (−2.3–23.8)
	Standard	20	20	0	
**Waist circumference, cm**					
<Median of 90/85 for male/female	Intensive	53.1	92.6	39.5 (10.4–50.8)	6.2 (−0.2–10.3)
	Standard	21.2	54.5	33.3 (7.7–46.9)	
≥Median of 90/85 for male/female	Intensive	43.4	63.2	19.8 (9.7–43.6)	19.8 (−8.6–31.4)
	Standard	16.7	16.7	0	
**TC, mmol/L**					
<Median of 5.2	Intensive	53.8	67.9	14.1 (−2.4–32.9)	7.4 (−1.2–14.7)
	Standard	13.3	20	6.7 (−10.9–21.9)	
≥Median of 5.2	Intensive	43	88.6	45.6 (32.8–61.7)	2.7 (−1.4–12.4)
	Standard	28.6	71.4	42.9 (27.4–59.0)	
**TG, mmol/L**					
<Median of 1.7	Intensive	51.2	73.2	21.9 (9.6–43.7)	1.4 (−1.2–9.4)
	Standard	17.9	38.5	20.5 (−7.2–30.8)	
≥Median of 1.7	Intensive	45.3	84	38.7 (16.8–53.4)	13.7 (−1.3–25.8)
	Standard	25	50	25.0 (3.8–37.4)	

Mixed models were used in the analyses to see how outcomes changed over time and how they were affected by interventions. The model included fixed effects for 2 groups, time of measurement, and the model were adjusted by some baseline characteristics such as age, gender, educational level, and so on. Statistically significant results are presented in bold.

## Discussion

Comprehensive intensive intervention of hypertension control carried out in Chinese communities has been proved effective, which can significantly improve the control rate of hypertension, and the hypertension prevention and control technologies are relatively mature^13^. This study showed that the comprehensive intensive intervention of occupational hypertension could also effectively control BP of college staff and improved the lifestyles. Intervention effects on hypertension control rate of some subgroups were better, including subgroups of that younger than 50 years old, male, university and above, drinking, and irregular exercise. After 2 years of intervention, compared with the standard intervention group, SBP/DBP in the intensive intervention group decreased by 3.7/4.0 mmHg and BP control rate increased by 8.9%. The results were on par or even better than the effects of renal denervation on management of resistant hypertension as showed by agasthi *et al*.^14^. This showed the importance and beneficial effect of comprehensive management of hypertension over the standard of care practiced in medicine.

The results of this study showed that smoking patients and drinking patients in the intensive intervention group decreased significantly, while fruit, vegetable consumption and regular exercise increased, which helped to improve the hypertension control^15^. It was indicated that the health promotion project in schools would make positive changes in the behaviors and lifestyles of employees, and it might be also helpful for other occupational population^16^. Intensive intervention including of follow-up management and health education made patients realize the importance of healthy lifestyle. And the employees’ behaviors improved effectively by creating a healthy supportive environment in the workplace. In this study, the intervention effect of BMI and waist circumference was not statistically significant, which was consistent with the conclusion of previous studies^17^. It was suggested that there was difficulty in weight management. We should carry out long-term health education based on the working environment, and advocate the concept of lifestyle therapy to improve life behaviors. Meanwhile, comprehensive intensive intervention including systematic and long-term follow-up management contributed to the improvement of behaviors and lifestyles, and helped hypertension control which needed lifelong treatment.

Subgroup analysis of hypertension control rates found that the effect of the intervention in male subgroup was most obvious. It might be because that male group had slightly worse living habits including smoking, drinking, less fruit and vegetables consumption and other problems before the intervention. Through the comprehensive intervention including follow-up management, lifestyles of male subgroup improved, which contributing to the BP control. Meanwhile, the intervention effects of the subgroups younger than 50 years old and having degree of university and above were effectively. It might be because that patients young and having higher education levels accepted intensive intervention quickly and improved lifestyles more effectively, who also had a higher compliance. In addition, the effect of intervention was better in drinking subgroup and irregular exercise subgroup. It might be that these factors were all adverse ones affecting hypertension, and the positive effect of BP control could be achieved by improving the lifestyles through intervention.

Increasing evidence suggested that non-pharmacological treatments, such as yoga and lifestyle modification helped to the hypertension control^18,19^. And emotional intervention had been used in the treatment of some diseases. For example, psychological intervention improved the prognosis of patients with cancer and decreased the occurrence of cardiovascular events^20,21^. After 2 years of intervention, the life quality score of the intensive intervention group was significantly improved, while that of the standard intervention group was not significantly changed, and the effect of the intensive intervention group was significantly better than that of the standard intervention group, especially for the relief of anxiety and tension. It was suggested that emotional and psychological intervention was helpful among employees in schools which would contribute to the hypertension control.

Repeated measurements from patients were likely to be more similar to each other, so we chose mixed models to solve the problem of correlation which needed to be considered in the analysis of the resulting data. Meanwhile, using mixed models, reasonably valid estimates of treatment effects could often be obtained even when the missing values were not completely random and additional methods for handling missing data, such as multiple imputation, were generally not required^22^.

This study had several strengths and limitations. The main strength was that we pay attention to the hypertension control intervention of occupational population, which filled the gap of the community population which used to be the main concern of hypertension control. Moreover, this study did not only provide a comprehensive intensive intervention to hypertension control, but also paid attention to the quality life of employees working in schools, especially to the emotional and psychological intervention. Meanwhile, we used the mixed effect model to analyze and evaluate the results, maximize the utility of data, and solve the correlation problem. Despite these strengths, there were several limitations. Firstly, the long-term effects such as the incidence of cardiovascular events needed to be further observed. Secondly, the sample size was relatively small, which leading to the sample sizes in the stratification analysis process of some subgroups were small. Moreover, some information was collected in the standard intervention group only at baseline and the 2th year in order to avoid confounding caused by follow-up, which leading to the information lack of the dynamic trend in the standard intervention group. Meanwhile, we only included the college staff population, other occupational populations should be considered for verification. Finally, we did not perform a follow up study on the effect of comprehensive intensive intervention on patients with resistant hypertension to see the implications on hypertension management. Therefore, a study with larger scale, larger sample size and longer period is needed and should conducted among more workplaces in the further. And sub-studies including of subjects with resistant hypertension should also be conducted.

The results of this study showed that comprehensive intensive intervention of hypertension control including follow-up management, emotional intervention and lifestyle intervention in occupational places of colleges and universities could improve patients’ behaviors and lifestyles, and significantly improved hypertension control rate. It was helpful to promote the development of the prevention, treatment and control of hypertension among staff in colleges and universities.

## Supplementary information

Spanish Hypertension Quality of Life Questionnaire (MINICHAL)
