# Supplementary material for: Effects of a comprehensive intervention on hypertension control in Chinese employees working in universities based on mixed models
Source: Sci Rep. 2019 Dec 16;9:19187. doi: 10.1038/s41598-019-55849-6 (PMC6914772; doi:10.1038/s41598-019-55849-6)
Supplement: Supplementary file 1 — Spanish Hypertension Quality of Life Questionnaire (MINICHAL) [file 41598_2019_55849_MOESM1_ESM.doc]

**Effects of a comprehensive intervention on hypertension control in Chinese employees working in universities based on mixed models**

YANG Li^1, 2, #^, JIN Xiaoqing^1, #^, TANG Xinhua^1^, SHOU Xiaoling^1^, XU Xiaoling^1^, YU Wei^1^, WANG Zengwu^3^, WANG Xin^3^, ZHENG Pinpin^2^, *, YAN Jing^1^, *

^1^ Zhejiang Provincial Center for Cardiovascular Disease Control and Prevention, Zhejiang Hospital, Hangzhou 310013, China

^2^ Key Laboratory of Public Health Safety, Ministry of Education, Health Communication Institute, Fudan University, 138 Yixueyuan Road, Shanghai 200032, China

^3^ Department of Community Prevention and Control, National Cardiovascular Disease Center, Fuwai Hospital, BeiJing 102308, China

^#^ These authors contributed equally to this work

* Correspondence and requests for materials should be addressed to J.Y. (phoebe84331@163.com) and P.Z. [(zpinpin@shmu.edu.cn)](mailto:(zpinpin@shmu.edu.cn))

Supplement 1. Spanish Hypertension Quality of Life Questionnaire (MINICHAL).

During the past 7 days… No, not at all. Yes, somewhat. Yes, a lot. Yes, very much.

1. Have you been sleeping poorly?

2. Have you had difficulty maintaining

your usual social relationships?

3.Have you had difficulty interacting

with other people?

4.Have you felt that you are not

playing a useful role in life?

5.Have you felt unable to make

decisions and start new things/projects?

6.Have you felt continuously distressed

and tense?

7.Have you felt that life is a constant

struggle?

8.Have you felt incapable of enjoying

your daily activities?

9. Have you felt worn-out and powerless?

10. Have you felt sick?

11. Have you had difficulty breathing

or felt breathless for no apparent reason?

12. Have your ankles been swollen?

13. Have you noticed that you are

urinating more frequently?

14. Has your mouth been dry?

15. Have you felt pain in the chest

without doing any physical exertion?

16. Have you noticed numbness or

a tingling sensation in any part of the body?

17.Would you say that your hypertension

and its treatment have affected your quality

of life?
